# Influenza-Related Mortality Trends in Japanese and American Seniors: Evidence for the Indirect Mortality Benefits of Vaccinating Schoolchildren

**DOI:** 10.1371/journal.pone.0026282

**Published:** 2011-11-07

**Authors:** Vivek Charu, Cécile Viboud, Lone Simonsen, Katharine Sturm-Ramirez, Masayoshi Shinjoh, Gerardo Chowell, Mark Miller, Norio Sugaya

**Affiliations:** 1 Fogarty International Center, National Institutes of Health, Bethesda, Maryland, United States of America; 2 Department of Global Health, School of Public Health and Health Services, George Washington University, Washington D.C., United States of America; 3 Department of Pediatrics, School of Medicine, Keio University, Tokyo, Japan; 4 School of Human Evolution and Social Change, Arizona State University, Arizona, United States of America; 5 Department of Pediatrics, Keiyu Hospital, Yokohama, Japan; University of Hong Kong, Hong Kong

## Abstract

**Background:**

The historical Japanese influenza vaccination program targeted at schoolchildren provides a unique opportunity to evaluate the indirect benefits of vaccinating high-transmitter groups to mitigate disease burden among seniors. Here we characterize the indirect mortality benefits of vaccinating schoolchildren based on data from Japan and the US.

**Methods:**

We compared age-specific influenza-related excess mortality rates in Japanese seniors aged ≥65 years during the schoolchildren vaccination program (1978–1994) and after the program was discontinued (1995–2006). Indirect vaccine benefits were adjusted for demographic changes, socioeconomics and dominant influenza subtype; US mortality data were used as a control.

**Results:**

We estimate that the schoolchildren vaccination program conferred a 36% adjusted mortality reduction among Japanese seniors (95%CI: 17–51%), corresponding to ∼1,000 senior deaths averted by vaccination annually (95%CI: 400–1,800). In contrast, influenza-related mortality did not change among US seniors, despite increasing vaccine coverage in this population.

**Conclusions:**

The Japanese schoolchildren vaccination program was associated with substantial indirect mortality benefits in seniors.

## Introduction

Despite current approaches to prevention and control, seasonal influenza remains a significant cause of morbidity and mortality worldwide. While the elderly have the highest mortality rates of all age groups, school-aged children are the most important sources of community-wide transmission [1]. Vaccination is currently the most effective means of preventing seasonal influenza. Until recently, the seasonal vaccination strategy in the US targeted persons at risk for serious complications including seniors aged ≥65 years, individuals with chronic conditions, children aged 6–23 months, and pregnant women [2]. Vaccine recommendations have gradually been expanded to include all persons ≥6 months, but vaccination coverage among school-aged children in the US has remained modest [3], [4]. Analysis of US national vital statistics revealed that increasing vaccination coverage in seniors from 1980 to 2001 did not correlate with a decline in influenza-related mortality [5]; influenza-related hospitalization rates increased steadily in persons aged ≥50 years during this time period [6]. These findings suggest that the strategy of vaccinating only “high risk” populations may not be sufficient to decrease influenza transmission and severe burden at the population level, in part due to the weak immune response of seniors to vaccination [7].

A complementary, albeit debated, vaccination strategy specifically targets schoolchildren to reduce community-wide transmission of seasonal influenza. School-based trials and observational studies in the US and Russia suggest that the vaccination of schoolchildren can reduce influenza-related morbidity and mortality among non-immunized contacts and the elderly [8]–[14]. In a recent cluster-randomized controlled trial, immunization of ∼80% of schoolchildren conferred a 61% indirect protection against influenza infection to unvaccinated individuals [15]. While promising, these studies were conducted in small or selected populations and do not necessarily provide sufficient evidence to change vaccination policies [1]. Further evaluation of the population-level effects of different vaccination strategies is warranted to improve influenza control programs.

The Japanese experience offers a unique opportunity to examine the population-level benefits of vaccinating schoolchildren. From 1977 to 1987, the Japanese schoolchildren vaccination program achieved between 50 and 85% annual coverage in children aged 3 to 15 years. The program was officially discontinued in 1994, when the priority groups for vaccination switched to seniors aged ≥65 years and those aged 60–64 years with high-risk conditions [16]–[21] (Table S1). A previous study of mortality trends by Reichert et al. showed that the Japanese schoolchildren vaccination program was associated with a decrease in the overall number of influenza-related excess deaths and that excess deaths increased once the program was discontinued [16]. Unfortunately, the study suffered from methodological issues related to the estimation of influenza disease burden, and did not adjust for variation in circulating strains and rapid socio-economic and demographic changes following World War II [22]–[24]. Further, Reichert et al. could not quantify the indirect benefits of schoolchildren vaccination on mortality among seniors due to lack of age-specific data [16]. In the present study, we carefully re-examine the Japanese vaccination strategy by analyzing detailed age-specific mortality and virus surveillance data, and address previous methodological criticisms. More importantly, we develop a statistical approach to quantify the indirect effects of schoolchildren vaccination on influenza-related mortality in seniors, using data from the US as a control.

## Methods

A detailed description of the data sources and analytic approach is given in Information S1 and summarized below.

### Mortality data and excess mortality approach

We compiled monthly pneumonia and influenza (P&I) deaths and population estimates for individuals ≥65 yrs during 1978–2006 from the Japanese Ministry of Health, Labor and Welfare and the US National Center for Health Statistics (Table S2). As in previous studies, we concentrated on trends in P&I mortality rates, a specific indicator of influenza-related mortality [25], [17]. We focused on the 1978–2006 time period to obtain reliable information on vaccine coverage ([17] and Table S1) and to avoid the period of adaptation following the 1968 influenza A/H3N2 pandemic, during which mortality in Japan and the US declined steeply even in the absence of vaccination [16]. Mortality data were stratified by five senior age groups (65–69, 70–74, 75–79, 80–84, 85–89 years) to allow careful adjustment for changing post-war demographics. Data on dominant influenza subtypes circulating in Japan and the US during the study period were obtained from [5], [17].

To estimate seasonal influenza-related mortality rates, we applied Serfling cyclical regression models to monthly P&I death rates for each country and age-group, as in [5], [26]. These models provide seasonal baseline levels of expected mortality in the absence of influenza virus circulation. Mortality observed in excess of the baseline during influenza-epidemic months is attributed to influenza and termed “seasonal excess mortality”.

### Estimating excess mortality reduction among seniors

To account for long-term changes in mortality unrelated to influenza and for baseline differences between Japan and the US, we standardized winter-seasonal excess P&I mortality rates for changes in population structure and summer mortality rates (Information S1). This approach has been used in the past to adjust for differences in socioeconomic status and access to healthcare between high and middle-income countries [27]. We chose the US summer P&I mortality rate and population structure in 2000 as a reference.

To quantify the indirect benefits of the Japanese vaccination program, we assessed differences in crude and adjusted excess P&I mortality rates during the schoolchildren vaccination period (1978–1994) and the period immediately after the program was discontinued (1995–2006). We modeled age-stratified seasonal excess death rates using multivariate negative binomial regression, using a log-link with a dummy indicator for time period, adjusting for age and dominant virus subtype. A formal description of the model used and model diagnostics are provided in the Fig. S3, Table S8. We checked that interaction terms between the time period and age variables were not significant in the model. We also compared influenza-related mortality trends in Japan with those in the US using the same methodology, utilizing the US experience as a concurrent control that did not implement the vaccination of schoolchildren.

## Results

### Influenza-related mortality trends in Japanese and American seniors

**Figure 1:**
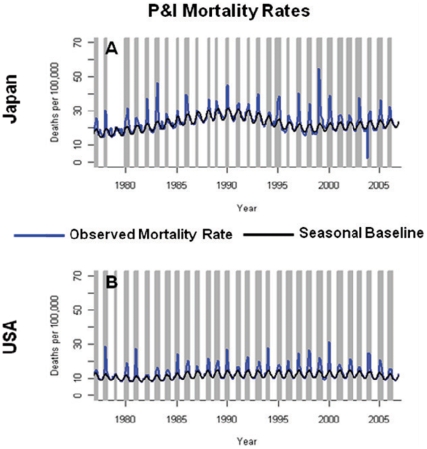
Pneumonia and influenza (P&I) mortality rates among the Japanese (A) and US (B) elderly, aged 65–89 years, Jan. 1977–Dec. 2006. Expected mortality rates in the absence of influenza virus circulation are shown in black, as determined by the Serfling seasonal baseline approach. Influenza epidemic months are highlighted in grey.

Monthly time series of P&I mortality rates in Japanese and American elderly, aged 65–89 years, reveals a series of peaks in November–April during 1978–2006 (Fig. 1). On average, peaks of P&I mortality in Japan reached magnitudes ∼50% larger than those in the US. Influenza A/H3N2 was dominant in 17 of the 29 seasons studied in both countries.

**Figure 2:**
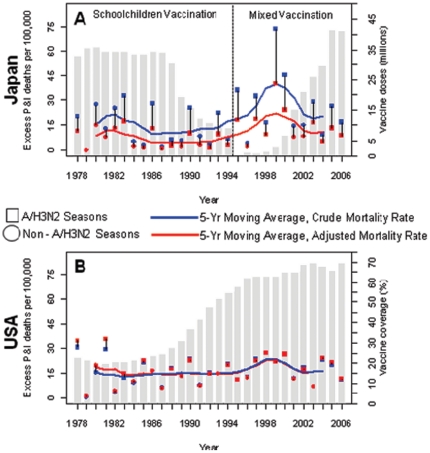
Seasonal excess pneumonia and influenza (P&I) mortality rates among the Japanese (A) and the US elderly (B), aged 65–89 years, 1977–78 to 2005–06 seasons. Squares represent seasons dominated by influenza A/H3N2 viruses; circles represent seasons dominated by influenza A/H1N1 or B. Blue symbols represent crude excess mortality estimates; red symbols represent excess mortality estimates adjusted for population aging and trends in baseline mortality risk. The blue and red lines represent 5-yr moving averages of the crude and adjusted seasonal excess mortality rates, respectively. Grey bars in (A) represent the number of vaccine doses distributed per influenza season in Japan; grey bars in (B) represent the influenza vaccine coverage among the US non-institutionalized elderly aged ≥65.

**Figure 3:**
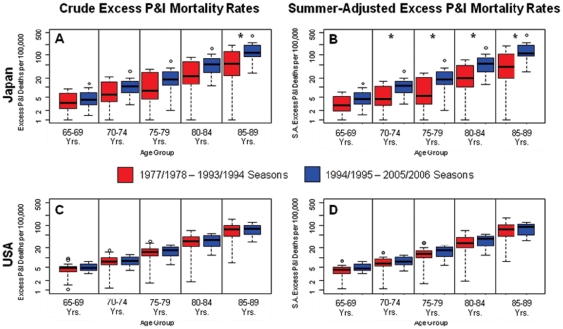
Crude (left panels) and adjusted (right panels) excess pneumonia & influenza (P&I) seasonal mortality rates by age group and time period in Japan (top) and the US (bottom). Rates were adjusted for time trends in the baseline risk of mortality. Asterisks indicate statistically significant differences between the two time periods using Wilcoxon's Rank Sum Test (P<0.05).

To examine long-term trends in influenza-related mortality we calculated excess P&I mortality rates for 29 winter seasons, 1977/1978 to 2005/2006 (Fig. 2, Table S3). On average, over the entire period studied, crude excess mortality rates were higher in Japan than in the US (19 v. 16 per 100000, respectively), but this patterns reversed after adjustment for population demographics and socio-economic trends (Table 1). Indeed, adjustment for between-country differences in socio-economic conditions, using summer mortality rates experienced in the US in 2000 as a reference, resulted in a ∼50% reduction in Japanese winter-seasonal excess P&I mortality rates. In both countries, excess mortality rates increased sharply with age (Fig. 3A & 3C, Tables S4) and were 2.1 to 2.5- fold higher during A/H3N2-dominant seasons than those dominated by influenza A/H1N1 or B (Table S3). A sharp elevation in excess mortality was observed in the US and Japan during 1997–2000, a period concurrent with four sequential A/H3N2-dominated seasons (Fig. 2).

**Table 1:** Average excess P&I mortality rates per 100,000 in Japanese and US seniors aged 65–89 yrs, 1978–2006.

	1978–2006*Avg. (SD)*	1978–1994*Avg. (SD)*	1995–2006*Avg. (SD)*	% change between time periods	P-value
**Japan Excess P&I**	19.18 (15.9)	13.85 (11.0)	26.73 (19.0)	93%	0.034
**Japan Adjusted Excess P&I**	10.00 (8.7)	6.82 (5.8)	14.51 (10.3)	113%	0.027
**USA Excess P&I**	16.39 (7.7)	15.26 (8.3)	17.99 (6.9)	18%	0.445
**USA Adjusted Excess P&I**	16.94 (8.5)	16.25 (9.5)	17.91 (7.0)	10%	0.527

Standard deviations are tabulated in parentheses. Percent changes were calculated as the mean mortality rate in 1995–2006 (the period after discontinuation of the Japanese schoolchildren vaccination program) minus that in 1978–1994 (the schoolchildren vaccination program period in Japan), divided by the mean mortality rate in 1978–1994. P-values were determined using Wilcoxon's Rank Sum Test. Adjusted estimates were standardized to the US population structure of 2000 and corrected for time trends in the baseline risk of mortality (see Methods).

### Estimating the indirect mortality benefits of the Japanese schoolchildren vaccination program

To evaluate the effectiveness of the Japanese schoolchildren vaccination program we compared excess influenza-related mortality rates when the program was in place (1978–1994) with those in the decade after the program was discontinued (1995–2006). In Japan, average crude and adjusted excess P&I mortality rates among seniors increased by 93 and 113%, respectively, during the post-schoolchildren vaccination program period (P<0.04, Table 1). In addition, excess mortality rates increased by 26 to 114% across the 5 senior age subgroups after the program was terminated (Fig. 3A & 3B, Tables S4). Interestingly, the increase in excess mortality rates was more pronounced in older age groups (P<0.05 for adjusted mortality rates among 70–89 year olds).

To check that the increase in excess P&I mortality in Japan after 1994 was specific to the discontinuation of the schoolchildren vaccination program, we used trends in excess P&I mortality in the US over the same time period as a control. In contrast to Japan, there was no difference in excess mortality rates before and after 1994, in any of the US senior age groups (Table 1, Fig. 3C–D). In addition, adjusted excess mortality rates in Japan were significantly lower than those in the US during the Japanese schoolchildren vaccination program (P = 0.001) and subsequently became similar to those in the US (P = 0.18, Table S5).

To further control for increased circulation of the influenza A/H3N2 subtype after the Japanese schoolchildren vaccination program was discontinued, we fit negative binomial models to seasonal excess mortality rates, with time period, dominant influenza subtype, and the five age groups as covariates (Table 2). In Japan, the relative risk of adjusted excess mortality in seniors was 0.64 during the vaccination program (95% CI: 0.49–0.83), corresponding to a 36% reduction in excess mortality through indirect protection (95% CI 17–51%). These vaccination benefits represent 992 adjusted excess deaths averted per season among seniors in Japan (95% CI: 355–1,825) or 7.5 deaths averted per 100,000 (95% CI: 2.8–14.4 per 100,000). The estimated benefits were lower in the unadjusted data (Table 2). In contrast to Japan, the same modeling approach applied to the US data revealed no significant difference in the risk of excess mortality between the two periods (Table 2).

**Table 2:** Relative risks of excess P&I death among seniors aged 65–89 in 1995–2006 compared to 1978–1994 in Japan and the US, controlling for influenza subtype dominance, population aging, and trends in baseline risk of mortality.

Model Outcome	Adjusted RR (1978–1994 v. 1995–2006)	Adjusted Protective Effectiveness of the Vaccination Program (%)	Number of Deaths Averted per Season due to the Schoolchildren Vaccination Program
Japan Adjusted Excess P&I:	0.64 (0.49–0.83)	35.90 (16.67–50.74)	992 (355–1825)
USA Adjusted Excess P&I:	1.04 (0.87–1.24)	−4.16 (−23.46–13.04)	_

The period 1978–1994 corresponds to the schoolchildren vaccination program in Japan, which was discontinued after 1994. Vaccine protective effectiveness estimates were calculated as (1-1/RR)×100.

To ensure that our findings were not simply the byproduct of the particular time periods chosen, we repeated our analysis using different time periods for trend comparisons. For instance, we compared the last ten influenza seasons of the schoolchildren vaccination program (1983–1994) with the first ten seasons after the program was discontinued (1995–2006). The reductions in excess P&I mortality rates associated with the schoolchildren vaccination program were even more pronounced when we considered these shorter periods (Table S7).

Finally, as a sensitivity analysis, we compared influenza vaccination strategies in Japan and the US by including excess mortality rates from both countries in the same model, allowing direct between-country comparisons (Table S6). This model confirmed that Japanese seniors were at lower risk of adjusted excess P&I death during the schoolchildren vaccination program compared to when the program was discontinued, and produced similar mortality reductions as before (36%, 95% CI: 17–50%). Interestingly, however, Japanese seniors remained at lower risk of death than US seniors during the entire study period (Table S6).

## Discussion

To evaluate the indirect mortality benefits of the Japanese schoolchildren vaccination program, we studied seasonal influenza-related excess mortality rates among Japanese and American seniors from 1978 to 2006. We found that crude excess P&I mortality rates in Japanese seniors increased by 93% after the schoolchildren vaccination program was discontinued in 1994. A large fraction of this increase was due to more frequent circulation of the severe influenza A/H3N2 virus subtype in recent years. After controlling for circulating subtype, trends in population demographics and baseline risk of death, we estimated that the schoolchildren vaccination program conferred a 36% protective benefit against influenza-related mortality to Japanese seniors (95% CI:17–51%). On average, we estimate that this program was associated with 992 excess P&I deaths averted per season (95% CI: 355–1,825). In addition to this statistical analysis, we developed an age-structured transmission model to estimate the indirect mortality benefits of schoolchildren vaccination (Information S1). The model predicted a 26–52% reduction in influenza-related mortality amongst senior populations, consistent with our statistical findings (Figs. S1–2).

To control for changes in the severity of circulating influenza viruses, we estimated trends in influenza-related mortality in the US, where influenza vaccination was targeted to high-risk groups and coverage among seniors has gradually increased to ∼65%. We did not find significant changes in influenza-related mortality rates among US seniors during the study period (1978–2006), consistent with previous work [16].

In 2001, Reichert et al. reported that the Japanese schoolchildren vaccination program prevented between 10,000 and 12,000 excess P&I deaths per season in the entire population, but the authors did not analyze mortality data specific to seniors [16]. Our estimate of 992 excess P&I deaths (95% CI: 335–1,825) averted among seniors is substantially lower. Reichert et al. estimated mortality reductions by comparing excess P&I mortality rates in 1990 to those in the 1960 s. Notably, influenza-related excess mortality rates in the 1950 s–60 s were declining sharply in all countries due to socio-economic changes. Excess mortality rates also declined during the years immediately following the 1968 A/H3N2 pandemic, likely due to the population-wide acquisition of natural immunity to these viruses over time (rather than to vaccination) [5], [28]. In addition, this study and others have shown that excess mortality estimates are very sensitive to the frequency of A/H3N2 virus circulation, which was not controlled for in the Reichert et al. study [16]. Therefore, we believe our evaluation of the schoolchildren vaccination program using mortality data specific to seniors, and including recent years for comparison, is more prudent and allows adjustment for important confounders.

Our results can also be compared to those of a recent Canadian cluster-randomized controlled trial, in which immunization of ∼80% of schoolchildren conferred 61% indirect protection against clinical influenza infection to unvaccinated individuals (95% CI: 8–83%) [15]. Our analysis suggests a lower indirect mortality benefit of 36% (95% CI: 17–51%), although the confidence intervals of the two studies are large and overlap. Our study's lower point estimate could be explained by lower vaccination rates among Japanese schoolchildren, or lower efficacy of historical vaccines [18], [19], [21]. We note that our age-structured transmission model corresponds well with the Canadian findings, predicting a ∼60% reduction in influenza-related mortality among seniors at high vaccine coverage (Fig. S2). Further research should focus on combining epidemiological data with mathematical transmission models to evaluate different vaccination strategies.

Several limitations of our study should be mentioned. We concentrated on P&I mortality, despite the fact that the impact of influenza is not limited to solely P&I [29]. The Japanese schoolchildren vaccination program was associated with a non-significant reduction in excess all-cause mortality in seniors (not shown), which could be explained by a lack of specificity and unaccounted baseline time trends [5], [25]. Although it would have been interesting to investigate trends in other causes of death traditionally linked to influenza, such as respiratory and cardiac diseases, these data were not available to us. Additionally, our study was ecological and prone to confounders, which is why we introduced the US comparison as a control. Furthermore, we adjusted for the circulation of more virulent A/H3N2 viruses, accounted for population aging by studying age-specific mortality rates and used sensitivity analyses to explore the robustness of our findings. We also supplemented our data on vaccine doses distributed with age-specific vaccine coverage data for selected years [18], [19], [21]. Finally, we used mortality data from summer periods, free of the effect of influenza, to adjust for time trends in the background risk of death among seniors in 1978–2006 due to differences in access to care, underlying co-morbidities or other causes. Patterns identified in adjusted excess mortality estimates were always in the same direction as those identified in crude mortality estimates.

We considered two competing explanations for the increase in excess influenza-related mortality observed in Japanese seniors after the schoolchildren vaccination program was discontinued: the growing impact of nursing homes and antiviral treatment. While the number of “Nursing Homes for the Elderly” increased 5 fold between 1980 and 2006 in Japan, the percentage of three-generation households decreased from 16.2 to 9.1% [30], [31]. However, the overall proportion of elderly persons living in nursing facilities remained low throughout the study period in Japan and could not account for the substantial increases seen in influenza-related mortality rates in recent years.

Oseltamivir use has increased substantially in Japan since 2003 in all age groups, making Japan the country with the highest annual level of oseltamivir use per capita, comprising >70% of the world's consumption in 2009 [32], [33]. The decline in influenza-related mortality in the Japanese elderly observed after 2000 may be due in part to the routine use of oseltamivir and in part to increasing vaccination rates amongst the elderly, other high-risk groups, and children aged 6–13 years (Fig. 2, Table S1). While it is too soon to precisely evaluate to effect of oseltamivir use on influenza-related mortality in the Japanese population, high oseltamivir usage was limited to the last 3 years of our study and would only bias our analyses towards null hypotheses.

In conclusion, numerous studies have reported that school-aged children have high influenza attack rates and play an important role in the transmission of influenza within schools, families and communities [34]–[38]. Importantly, children respond well to vaccination and vaccinating this age-group is cost-effective, regardless of indirect benefits to unvaccinated contacts [39]–[41]. Here, we have shown that the Japanese schoolchildren vaccination program was associated with significant reductions in influenza-related excess P&I mortality among the Japanese elderly. Our estimates of indirect vaccination benefits are conservative because they focus on P&I mortality, a fraction of the total mortality burden of influenza and because the Japanese population has not entirely escaped influenza vaccination or antiviral treatment since the schoolchildren vaccination program was discontinued. Our findings fit well with an accumulating body of theoretical and experimental evidence suggesting that high vaccination coverage of children can contribute to reductions in morbidity and mortality among non-immunized community members [42]–[44].

While the societal structure of Japan is markedly different from that of the US, we believe that our findings can extend to the US population for several reasons. First, several community-scale studies in the US have indicated that vaccinating schoolchildren against influenza confers herd immunity in unvaccinated community members [8]–[10], [15]. Second, the introduction of several pediatric vaccines has produced substantial herd protective effects on the population level, most notably the pneumococcal vaccine [34]. In particular, the US-introduction of a vaccine targeting seven types of pneumococcal disease in young children in 2000 has substantially reduced the rates of carriage and invasive disease amongst people aged >50 years [44]. Our findings support vaccination of school-aged children, a group included in the most recent ACIP recommendations, which encourage yearly seasonal influenza vaccination for all persons aged ≥6 months in the US [3]. While seasonal influenza vaccine coverage remained low in US children aged 6 months – 17 years during the 2008–2009 epidemic season, they reached ∼44% towards the end of 2009 [45], [46]. It will be interesting to compare influenza-related disease trends in the US with those in other countries as vaccine coverage continues to increase in pediatric populations.

## Supporting Information

Figure S1 **Schematic of the compartmental influenza transmission model used to evaluate observed trends in Japanese excess mortality.** Note that the actual model is structured into 15 age groups.
(DOC)

Figure S2 **Reduction in influenza-related mortality rates among Japanese elderly, results from an age-structured model of influenza transmission.** (A) Reduction in influenza-related mortality rates amongst the elderly (blue-red), as a function of influenza vaccine coverage in schoolchildren (y-axis) and effective reproduction number, R_e_, (x-axis) as predicted by our influenza transmission model. Vaccine efficacy is set at 42% (B) Same as in A) but with varying vaccine efficacy (y-axis); vaccination coverage in schoolchildren is held at 70%.
(DOC)

Figure S3 **Negative binomial model diagnostics.** Panel A depicts the model residuals v. predicted values, while panel B depicts the observed data v. the model fitted values (y = x line present for reference). See Table 2 of the main text for model results and interpretation; see Eq. 2 above for a full description of the statistical model.
(DOC)

Table S1 **Age-specific influenza vaccination rates, Japan, 2000–2006.**
(DOC)

Table S2 **Underlying cause of death codes used to identify mortality due to pneumonia and influenza (P&I: Pneumonia and Influenza, ICD: International Classification of diseases).**
(DOC)

Table S3 **Crude and adjusted influenza-related excess mortality rates per 100,000 among Japanese and American seniors aged 65–89 yrs, and dominant influenza subtypes in circulation, 1978–2006.** Adjusted mortality rates, standardized to the US population structure of 2000 and adjusted for time trends in the baseline risk of mortality, are displayed in parentheses.
(DOC)

Table S4 **Comparison of crude and adjusted excess P&I mortality rates per 100,000 among Japanese and US seniors in 1978–1994 with those in 1995–2006.** Adjusted rates take into account time trends in baseline mortality risk. Percent changes were calculated as the mean mortality rate in 1995–2006 minus that in 1978–1994, divided by the mean mortality rate in 1978–1994. P-values were determined using Wilcoxon's Rank Sum Test.
(DOC)

Table S5 **Comparison of adjusted excess P&I mortality rates per 100,000 between Japanese and American seniors, aged 65–89.** Standard deviations are tabulated in parentheses. P-values were determined using Wilcoxon's Rank Sum Test.
(DOC)

Table S6 **Sensitivity analysis including US and Japan mortality data in the same model.** We modeled age-adjusted excess P&I estimates in Japan and the USA using multivariate negative binomial regression. Adjusted excess P&I estimates were standardized to the US summer mortality rate of 2000. We evaluated three different vaccination periods (Japan 1978–1994, schoolchildren vaccination; Japan 1995–2006, mixed vaccination; USA 1978–2006, elderly vaccination), adjusting for age, time trends in baseline mortality risk, and A/H3N2 subtype dominance.
(DOC)

Table S7 **Sensitivity analysis of time periods considered in mortality trend comparisons.** Comparison of crude and adjusted excess P&I mortality rates among Japanese and US seniors in 1983–1994 with those in 1995–2006. Adjusted rates take into account time trends in baseline mortality risk. Percent changes were calculated as the mean mortality rate in 1995–2006 minus that in 1983–1994, divided by the mean mortality rate in 1983–1994. P-values were determined using Wilcoxon's Rank Sum Test.
(DOC)

Table S8 **Parameter estimates and standard errors of the negative binomial model used to estimate the reduction in influenza-related mortality among Japanese seniors during the vaccination of schoolchildren time period (1978–1994).** Note that the 65–69 year old age group is used as a reference, and all model terms are statistically significant (P<0.05). See Eq. 2 above for a full description of the statistical model.
(DOC)

Information S1 **Supplemental methods.**
(DOC)
