# Supplementary material for: Influenza-Related Mortality Trends in Japanese and American Seniors: Evidence for the Indirect Mortality Benefits of Vaccinating Schoolchildren
Source: PLoS One. 2011 Nov 7;6(11):e26282. doi: 10.1371/journal.pone.0026282 (PMC3210121; doi:10.1371/journal.pone.0026282)
Supplement: Figure S1 — Schematic of the compartmental influenza transmission model used to evaluate observed trends in Japanese excess mortality. Note that the actual model is structured into 15 age groups. (DOC) [file pone.0026282.s001.doc]

**
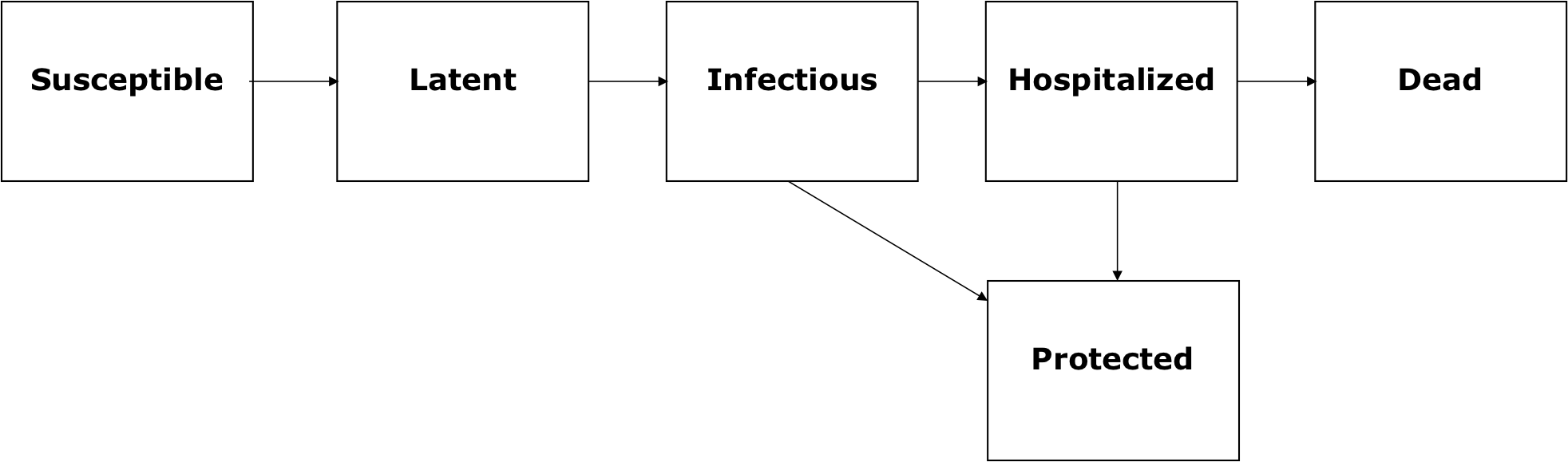
**

**Figure S1.** **Schematic of the compartmental influenza transmission model used to evaluate observed trends in Japanese excess mortality.** Note that the actual model is structured into 15 age groups.
