# Supplementary material for: Influenza-Related Mortality Trends in Japanese and American Seniors: Evidence for the Indirect Mortality Benefits of Vaccinating Schoolchildren
Source: PLoS One. 2011 Nov 7;6(11):e26282. doi: 10.1371/journal.pone.0026282 (PMC3210121; doi:10.1371/journal.pone.0026282)
Supplement: Figure S2 — Reduction in influenza-related mortality rates among Japanese elderly, results from an age-structured model of influenza transmission. (A) Reduction in influenza-related mortality rates amongst the elderly (blue-red), as a function of influenza vaccine coverage in schoolchildren (y-axis) and effective reproduction number, Re, (x-axis) as predicted by our influenza transmission model. Vaccine efficacy is set at 42% (B) Same as in A) but with varying vaccine efficacy (y-axis); vaccination coverage in schoolchildren is held at 70%. (DOC) [file pone.0026282.s002.doc]

**
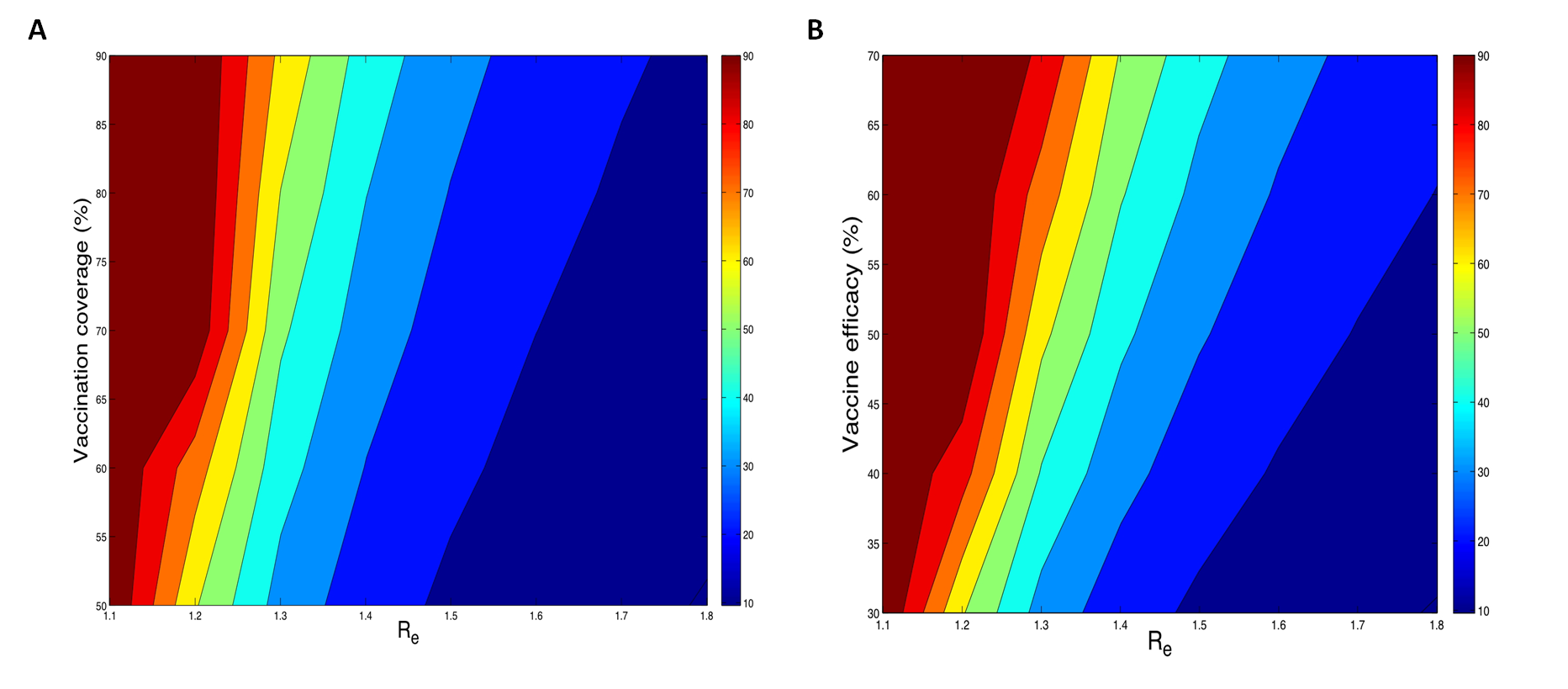
**

**Figure S2. Reduction in influenza-related mortality rates among Japanese elderly, results from an age-structured model of influenza transmission.** (A) Reduction in influenza-related mortality rates amongst the elderly (blue-red), as a function of influenza vaccine coverage in schoolchildren (y-axis) and effective reproduction number, R_e_, (x-axis) as predicted by our influenza transmission model. Vaccine efficacy is set at 42% (B) Same as in A) but with varying vaccine efficacy (y-axis); vaccination coverage in schoolchildren is held at 70%.
