# Supplementary material for: Influenza-Related Mortality Trends in Japanese and American Seniors: Evidence for the Indirect Mortality Benefits of Vaccinating Schoolchildren
Source: PLoS One. 2011 Nov 7;6(11):e26282. doi: 10.1371/journal.pone.0026282 (PMC3210121; doi:10.1371/journal.pone.0026282)
Supplement: Figure S3 — Negative binomial model diagnostics. Panel A depicts the model residuals v. predicted values, while panel B depicts the observed data v. the model fitted values (y = x line present for reference). See Table 2 of the main text for model results and interpretation; see Eq. 2 above for a full description of the statistical model. (DOC) [file pone.0026282.s003.doc]

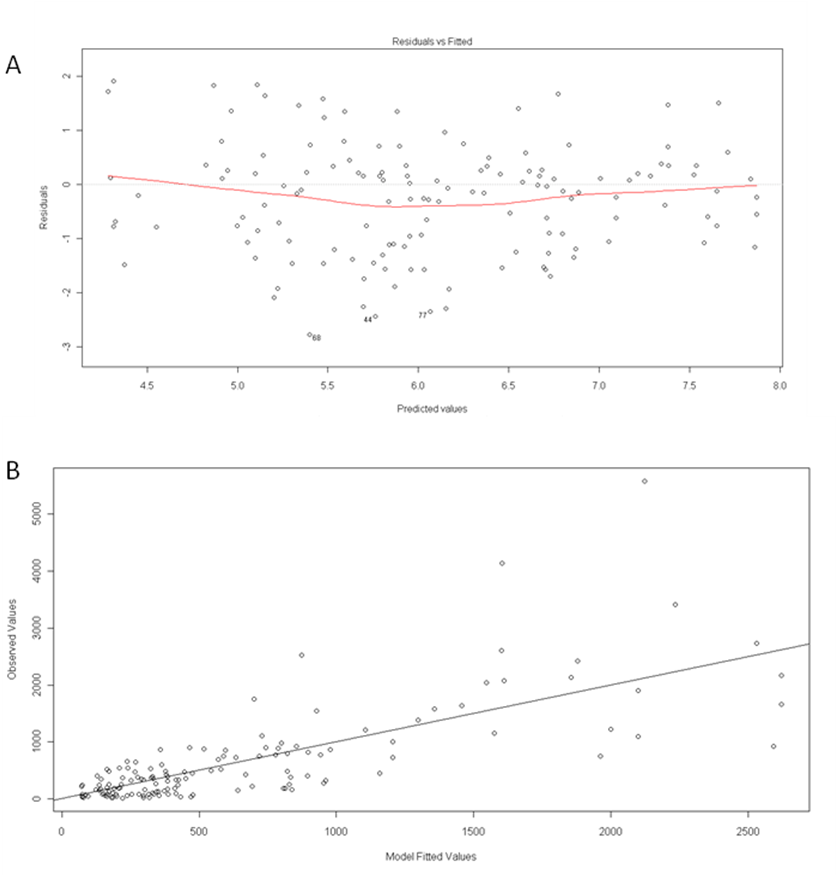


**Figure S3.** **Negative binomial model diagnostics.** Panel A depicts the model residuals v. predicted values, while panel B depicts the observed data v. the model fitted values (y = x line present for reference). See Table 2 of the main text for model results and interpretation; see Eq. 2 above for a full description of the statistical model.
