# Supplementary material for: Influenza-Related Mortality Trends in Japanese and American Seniors: Evidence for the Indirect Mortality Benefits of Vaccinating Schoolchildren
Source: PLoS One. 2011 Nov 7;6(11):e26282. doi: 10.1371/journal.pone.0026282 (PMC3210121; doi:10.1371/journal.pone.0026282)
Supplement: Table S1 — Age-specific influenza vaccination rates, Japan, 2000–2006. (DOC) [file pone.0026282.s004.doc]

**Table S1. Age-specific influenza vaccination rates, Japan, 2000-2006.**

| **Year** | **Vaccination Rate in 1-6 Year Olds (%)** | **Vaccination Rate in 6-13 Year Olds (%)** | | **Vaccination Rate in 13-65 Year Olds (%)** | **Vaccination Rate 65+ Year Olds (%)** | **Vaccination in Population (%)** |
| --- | --- | --- | --- | --- | --- | --- |
| **2000** | 20.1 | | 6.0 | 3.9 | 17.2 | 8.0 |
| **2001** | 24.3 | | 11.0 | 5.2 | 33.9 | 11.7 |
| **2002** | 31.1 | | 16.2 | 7.1 | 39.1 | 14.9 |
| **2003** | 42.1 | | 24.8 | 12.6 | 48.1 | 21.6 |
| **2004** | 52.9 | | 31.2 | 14.5 | 48.2 | 23.9 |
| **2005** | 60.5 | | 40.4 | 19.6 | 53.1 | 29.4 |
| **2006** | 51.8 | | 37.0 | 18.4 | 51.3 | 28 |
