# Supplementary material for: Influenza-Related Mortality Trends in Japanese and American Seniors: Evidence for the Indirect Mortality Benefits of Vaccinating Schoolchildren
Source: PLoS One. 2011 Nov 7;6(11):e26282. doi: 10.1371/journal.pone.0026282 (PMC3210121; doi:10.1371/journal.pone.0026282)
Supplement: Table S2 — Underlying cause of death codes used to identify mortality due to pneumonia and influenza (P&I: Pneumonia and Influenza, ICD: International Classification of diseases). (DOC) [file pone.0026282.s005.doc]

**Table S2. Underlying cause of death codes used to identify mortality due to pneumonia and influenza** (P&I: Pneumonia and Influenza, ICD: International Classification of diseases).

| **Year** | **ICD Version** | **P&I Codes** |
| --- | --- | --- |
| 1977-1979 | ICD-8 | 470-474 & 480-486 |
| 1980-1999 | ICD-9 | 480-487 |
| 2000-2006 | ICD-10 | J10-J18 |
