# Supplementary material for: Influenza-Related Mortality Trends in Japanese and American Seniors: Evidence for the Indirect Mortality Benefits of Vaccinating Schoolchildren
Source: PLoS One. 2011 Nov 7;6(11):e26282. doi: 10.1371/journal.pone.0026282 (PMC3210121; doi:10.1371/journal.pone.0026282)
Supplement: Table S3 — Crude and adjusted influenza-related excess mortality rates per 100,000 among Japanese and American seniors aged 65–89 yrs, and dominant influenza subtypes in circulation, 1978–2006. Adjusted mortality rates, standardized to the US population structure of 2000 and adjusted for time trends in the baseline risk of mortality, are displayed in parentheses. (DOC) [file pone.0026282.s006.doc]

**Table S3. Crude and adjusted influenza-related excess mortality rates per 100,000 among Japanese and American seniors aged 65-89 yrs, and dominant influenza subtypes in circulation, 1978-2006.** Adjusted mortality rates, standardized to the US population structure of 2000 and adjusted for time trends in the baseline risk of mortality, are displayed in parentheses.

| **Year** | **Excess P&I, Japan** | **Excess P&I, USA** | **Dominant Subtype, Japan** | **Dominant Subtype, USA** |
| --- | --- | --- | --- | --- |
| 1978 | 20.21 (11.38) | 30.5 (34.53) | A(H3N2) | A(H3N2) |
| 1979 | 0 (0) | 0.77 (0.94) | A(H1N1) | A(H1N1) |
| 1980 | 27.4 (15.27) | 15.61 (19.68) | A(H1N1) | B |
| 1981 | 12.72 (7.72) | 29.18 (35.87) | A(H1N1) | A(H3N2) |
| 1982 | 25.2 (13.52) | 3.54 (4.08) | B | B |
| 1983 | 32.74 (17.52) | 12.53 (14.65) | A(H3N2) | A(H3N2) |
| 1984 | 5.36 (2.42) | 9.41 (10.21) | A(H1N1) | A(H1N1), B |
| 1985 | 3.25 (1.35) | 21.38 (22.53) | B | A(H3N2) |
| 1986 | 27.73 (12.79) | 16.59 (16.57) | A(H3N2) | B |
| 1987 | 1.94 (0.94) | 6.53 (6.28) | A(H1N1) | A(H1N1) |
| 1988 | 6.22 (2.68) | 17.89 (17.67) | A(H3N2) | A(H3N2) |
| 1989 | 5.53 (2.27) | 13.66 (13.38) | A(H1N1) | B, A(H1N1) |
| 1990 | 25.79 (10.19) | 23.52 (23.04) | A(H3N2) | A(H3N2) |
| 1991 | 8.52 (3.4) | 7.79 (7.63) | A(H1N1) | B |
| 1992 | 3.59 (1.56) | 14.95 (14.53) | A(H3N2) | A(H3N2) |
| 1993 | 22.41 (9.82) | 14.82 (14.55) | A(H3N2) | B |
| 1994 | 6.83 (3.10) | 20.77 (20.18) | A(H3N2) | A(H3N2) |
| 1995 | 36.03 (18.49) | 11.26 (11.04) | A(H3N2) | A(H3N2) |
| 1996 | 4.04 (2.29) | 12.74 (12.43) | A(H1N1) | A(H1N1) |
| 1997 | 32.55 (18.93) | 23.23 (22.45) | A(H3N2) | A(H3N2) |
| 1998 | 16.74 (9.32) | 27.69 (27.44) | A(H3N2) | A(H3N2) |
| 1999 | 73.53 (40.28) | 22.46 (22.22) | A(H3N2) | A(H3N2) |
| 2000 | 45.41 (24.27) | 26.69 (26.67) | A(H3N2) | A(H3N2) |
| 2001 | 14.62 (7.86) | 11.75 (11.42) | A(H1N1) | A(H1N1), B |
| 2002 | 14.95 (8.31) | 18.22 (17.35) | A(H1N1) | A(H3N2) |
| 2003 | 29.46 (16.68) | 7.02 (6.87) | A(H3N2) | A(H1N1), B |
| 2004 | 9.73 (5.27) | 23.42 (24.17) | A(H3N2) | A(H3N2) |
| 2005 | 26.56 (13.49) | 20.13 (21.49) | A(H3N2) | A(H3N2) |
| 2006 | 17.13 (8.87) | 11.28 (11.4) | A(H3N2) | A(H3N2) |
