# Supplementary material for: Influenza-Related Mortality Trends in Japanese and American Seniors: Evidence for the Indirect Mortality Benefits of Vaccinating Schoolchildren
Source: PLoS One. 2011 Nov 7;6(11):e26282. doi: 10.1371/journal.pone.0026282 (PMC3210121; doi:10.1371/journal.pone.0026282)
Supplement: Table S5 — Comparison of adjusted excess P&I mortality rates per 100,000 between Japanese and American seniors, aged 65–89. Standard deviations are tabulated in parentheses. P-values were determined using Wilcoxon's Rank Sum Test. (DOC) [file pone.0026282.s008.doc]

**Table S5. Comparison of adjusted excess P&I mortality rates per 100,000 between Japanese and American seniors, aged 65-89.** Standard deviations are tabulated in parentheses. P-values were determined using Wilcoxon’s Rank Sum Test.

| **Adjusted Excess P&I Mortality Rates** | | | | |
| --- | --- | --- | --- | --- |
|  | **Japan** | **USA** | **Percent Difference** | **P-value** |
| **1978-2006** | 10.00 (8.7) | 16.94 (8.5) | 69.40 | 0.001 |
| **1978-1994** | 6.82 (5.8) | 16.25 (9.5) | 138.30 | 0.001 |
| **1995-2005** | 14.51 (10.3) | 17.91 (7.0) | 23.43 | 0.18 |
