# Supplementary material for: Influenza-Related Mortality Trends in Japanese and American Seniors: Evidence for the Indirect Mortality Benefits of Vaccinating Schoolchildren
Source: PLoS One. 2011 Nov 7;6(11):e26282. doi: 10.1371/journal.pone.0026282 (PMC3210121; doi:10.1371/journal.pone.0026282)
Supplement: Table S6 — Sensitivity analysis including US and Japan mortality data in the same model. We modeled age-adjusted excess P&I estimates in Japan and the USA using multivariate negative binomial regression. Adjusted excess P&I estimates were standardized to the US summer mortality rate of 2000. We evaluated three different vaccination periods (Japan 1978–1994, schoolchildren vaccination; Japan 1995–2006, mixed vaccination; USA 1978–2006, elderly vaccination), adjusting for age, time trends in baseline mortality risk, and A/H3N2 subtype dominance. (DOC) [file pone.0026282.s009.doc]

**Table S6. Sensitivity analysis including US and Japan mortality data in the same model.** We modeled age-adjusted excess P&I estimates in Japan and the USA using multivariate negative binomial regression. Adjusted excess P&I estimates were standardized to the US summer mortality rate of 2000. We evaluated three different vaccination periods (Japan 1978-1994, schoolchildren vaccination; Japan 1995-2006, mixed vaccination; USA 1978-2006, elderly vaccination), adjusting for age, time trends in baseline mortality risk, and A/H3N2 subtype dominance.

| **Comparison** | **Adjusted Relative Risk (95% CI)** |
| --- | --- |
| Japan 1978-1994 v. Japan 1995-2006: | 0.64 (0.50 -0.83) |
| Japan 1978-1994 v. USA 1978-2006: | 0.49 (0.40 -0.59) |
| Japan 1995-2006 v. USA 1978-2006: | 0.76 (0.60 -0.96) |
