# Supplementary material for: Influenza-Related Mortality Trends in Japanese and American Seniors: Evidence for the Indirect Mortality Benefits of Vaccinating Schoolchildren
Source: PLoS One. 2011 Nov 7;6(11):e26282. doi: 10.1371/journal.pone.0026282 (PMC3210121; doi:10.1371/journal.pone.0026282)
Supplement: Table S7 — Sensitivity analysis of time periods considered in mortality trend comparisons. Comparison of crude and adjusted excess P&I mortality rates among Japanese and US seniors in 1983–1994 with those in 1995–2006. Adjusted rates take into account time trends in baseline mortality risk. Percent changes were calculated as the mean mortality rate in 1995–2006 minus that in 1983–1994, divided by the mean mortality rate in 1983–1994. P-values were determined using Wilcoxon's Rank Sum Test. (DOC) [file pone.0026282.s010.doc]

**Table S7. Sensitivity analysis of time periods considered in mortality trend comparisons.** Comparison of crude and adjusted excess P&I mortality rates among Japanese and US seniors in 1983-1994 with those in 1995-2006. Adjusted rates take into account time trends in baseline mortality risk. Percent changes were calculated as the mean mortality rate in 1995-2006 minus that in 1983-1994, divided by the mean mortality rate in 1983-1994. P-values were determined using Wilcoxon’s Rank Sum Test.

|  | **Crude Excess P&I Mortality Rates** | | | | | **Adjusted Excess P&I Mortality Rates** | | | | |
| --- | --- | --- | --- | --- | --- | --- | --- | --- | --- | --- |
| **Age Group** | **1983-2006** | **1983-1994** | **1995-2006** | **Percent Change** | **P-Value** | **1983-2006** | **1983-1994** | **1995-2006** | **Percent Change** | **P-Value** |
| Japan |  |  |  |  |  |  |  |  |  |  |
| 65-69 | 3.44 | 2.72 | 4.16 | 52.81 | 0.27 | 3.19 | 2.08 | 4.30 | 107.37 | 0.05 |
| 70-74 | 9.16 | 7.018 | 11.29 | 60.87 | 0.14 | 8.27 | 4.88 | 11.66 | 138.74 | 0.02 |
| 75-79 | 16.86 | 11.53 | 22.18 | 92.37 | 0.08 | 15.15 | 7.87 | 22.43 | 184.86 | 0.02 |
| 80-84 | 45.79 | 32.04 | 59.53 | 85.80 | 0.06 | 43.49 | 24.97 | 62.00 | 148.30 | 0.01 |
| 85-89 | 105.30 | 65.12 | 145.50 | 123.43 | 0.02 | 102.10 | 55.82 | 148.30 | 165.68 | 0.003 |
|  |  |  |  |  |  |  |  |  |  |  |
| USA |  |  |  |  |  |  |  |  |  |  |
| 65-69 | 3.83 | 3.50 | 4.16 | 18.78 | 0.48 | 3.59 | 3.09 | 4.09 | 32.12 | 0.13 |
| 70-74 | 6.88 | 6.72 | 7.04 | 4.78 | 0.71 | 6.21 | 5.74 | 6.68 | 16.32 | 0.41 |
| 75-79 | 14.79 | 14.15 | 15.42 | 8.98 | 0.59 | 13.30 | 11.81 | 14.80 | 25.32 | 0.22 |
| 80-84 | 33.22 | 32.07 | 34.36 | 7.14 | 0.76 | 31.89 | 29.24 | 34.54 | 18.13 | 0.38 |
| 85-89 | 76.12 | 75.05 | 77.19 | 2.85 | 0.76 | 75.20 | 70.13 | 80.27 | 14.46 | 0.38 |
