# Supplementary material for: Influenza-Related Mortality Trends in Japanese and American Seniors: Evidence for the Indirect Mortality Benefits of Vaccinating Schoolchildren
Source: PLoS One. 2011 Nov 7;6(11):e26282. doi: 10.1371/journal.pone.0026282 (PMC3210121; doi:10.1371/journal.pone.0026282)
Supplement: Table S8 — Parameter estimates and standard errors of the negative binomial model used to estimate the reduction in influenza-related mortality among Japanese seniors during the vaccination of schoolchildren time period (1978–1994). Note that the 65–69 year old age group is used as a reference, and all model terms are statistically significant (P<0.05). See Eq. 2 above for a full description of the statistical model. (DOC) [file pone.0026282.s011.doc]

**Table S8. Parameter estimates and standard errors of the negative binomial model used to estimate the reduction in influenza-related mortality among Japanese seniors during the vaccination of schoolchildren time period (1978-1994).** Note that the 65-69 year old age group is used as a reference, and all model terms are statistically significant (P<0.05). See Eq. 2 above for a full description of the statistical model.

| **Parameter** | **Estimate** | **Std. Error** | **z-score** | **P-value** |
| --- | --- | --- | --- | --- |
| β_0_ (Intercept) | -10.8958 | 0.1712 | -63.643 | 2.00E-16 |
| β_1_ (Vaccination period) | 0.4464 | 0.1359 | 3.285 | 0.00102 |
| β_2_ (A/H3N2 dominance) | 0.5906 | 0.1378 | 4.286 | 1.82E-05 |
| β_3_(85-89 years) | 3.3732 | 0.206 | 16.376 | 2.00E-16 |
| β_4_(80-84 years) | 2.5188 | 0.206 | 12.228 | 2.00E-16 |
| β_5_(75-79 years) | 1.509 | 0.2061 | 7.323 | 2.43E-13 |
| β_6_(70-74 years) | 0.8876 | 0.2061 | 4.306 | 1.67E-05 |
