# Supplementary material for: Influenza-Related Mortality Trends in Japanese and American Seniors: Evidence for the Indirect Mortality Benefits of Vaccinating Schoolchildren
Source: PLoS One. 2011 Nov 7;6(11):e26282. doi: 10.1371/journal.pone.0026282 (PMC3210121; doi:10.1371/journal.pone.0026282)
Supplement: Information S1 — Supplemental methods. (DOC) [file pone.0026282.s012.doc]

**Supplementary Methods**

Mortality Data:

We extracted monthly pneumonia and influenza (P&I) deaths during 1977-2006 from the Ministry of Health, Labor and Welfare of Japan. Table S2 lists the *International Classification of Diseases* (ICD) codes used to identify P&I deaths. For each year, we generated summary datasets of the monthly numbers of P&I deaths for specific age intervals (all ages, under 4, 5-9, 10-14, 65-69, 70-74, 75-79, 80-84, 85-89 years).

We applied a similar approach to compile vital statistics for the US, using National Center for Health Statistics data over the same period. To adjust for the transition from ICD-9 to ICD-10 in 1999, we multiplied the reported number of monthly P&I deaths in the US during the 1999-2006 period by a correction factor of 0.6982 [1].

We elected to study P&I mortality after 1978 for two reasons. First, the schoolchildren vaccination program was mandated by law in 1977, and reliable vaccine coverage data are available only after the 1977-1978 influenza season. Second, and more importantly, we sought to avoid the post-1968 pandemic period of adaptation, during which mortality in Japan declined steeply even in the absence of vaccination.

Population Data:

We obtained data on the midyear populations of Japan from 1977-2006 from *The Vital Statistics of Japan*. We obtained annual population estimates for the US from the US Bureau of the Census by single year of birth for the years 1977-2006, and calculated the population of people in each age group. For both countries, we calculated monthly mortality rates per 100000 for each age group and standardized these to 30.4-day months.

Vaccine Usage Data:

Data on the amount of influenza vaccine distributed in Japan from 1978-2003 were obtained from Sugaya et al [2]. Similar data from 2004-2006 were supplied by the Association of Manufacturers of Biologic Products in Japan.

Japan:

From 1978-1987 in Japan, influenza vaccination for schoolchildren was mandatory by law. During this time, ~35 million vaccines were distributed per year (Fig. 2A). In 1987 vaccination was no longer obligatory, and the doses of vaccine distributed dropped sharply. In 1994, when the program was terminated, the number of vaccine doses distributed fell to less than 5 million. From 1995-2006, the elderly and other high-risk groups were targeted for vaccination; vaccine doses distributed to these populations have increased steadily during this time period, up to ~ 40 million doses in 2006.

US:

Vaccination rates in the US non-institutionalized elderly (aged ≥ 65) from 1978-2001 were obtained from Simonsen et al. [3]; similar data from 2002-2006 were obtained from the National Health Interview Survey (NHIS).

From 1978-2006, high-risk groups were targeted for influenza vaccination in the US. The vaccine coverage among the non-institutionalized elderly (aged ≥ 65) increased steadily during this time period, from 21% in 1978 to 70% in 2006 (Fig. 2B).

Influenza Virus Surveillance Data:

Data on the dominant influenza subtype circulating in Japan during 1978-2002 were obtained from Sugaya et al. [2]. Similar data for 2003-2006 were obtained from the Annual Report of National Epidemiological Surveillance of Vaccine-Preventable Diseases.

Data on the dominant influenza subtype circulating in the US during 1978-2001 were obtained from Simonsen et al. [3]. For 2002-2006, we reviewed annual *Morbidity and Mortality Weekly Report* influenza summaries of viral subtypes that were identified in US laboratories during each influenza season; we considered an influenza subtype to be dominant when it accounted for at least 50% of all isolates in that season.

Calculating Excess Mortality Rates:

To estimate seasonal excess P&I mortality rates, we applied a Serfling-type cyclical regression model to monthly death rates from 1978-2006 for each country and age-group, as described by Viboud et al. [4] and Simonsen et al. [3]. Briefly, for each age group, we first de-trended the time series by dividing it by a cubic spline smooth function fit to the average summer mortality rate (July-September) for each year. In order to obtain a model baseline for monthly P&I mortality rates, we fit a cyclical regression model to monthly mortality rates in May-November months, when the circulation of influenza viruses is low. The model was of the form:

Y­­_t_ = β_0_ + β_1_t + β_2_t^2^ + β_3_t^3^ + β_4_t^4^ + β_5_t^5^ + β_6_sin(2πt/12) + β_7_cos(2πt/12) + ε_t_

where Y_t_ denotes observed monthly mortality rates at month t, β_0_ represents the intercept, β_1_ represents a coefficient for the linear time trend, t, β_2_ represents a coefficient for the quadratic time trend, t^2^, β_3_ – β_5_ represent coefficients for other polynomial time trends, β_6_ and β_7_ represent coefficients associated with seasonal fluctuations in mortality rates, and ε_t_ represents the error term. In some instances, a logarithmic transformation of Y_t_ was necessary to correct for unequal error variances and non-normality of error terms. All parameters were estimated using the least-squares method. In all cases, best-fit models minimized Akaike’s Information Criterion (AIC) in a backwards variable selection procedure. All model terms included were statistically significant (P < 0.01).

Influenza epidemic periods were identified by applying this procedure to P&I mortality rates for the entire population. Winter months (November-April) in which observed P&I mortality rates exceeded the upper 95% confidence limit of the model baseline were termed “epidemic months”. Monthly excess P&I mortality rates were calculated as the observed mortality rate minus the predicted mortality rate in each epidemic month. Seasonal excess mortality rates were calculated based on the sum of excess mortality rates during each influenza epidemic month.

Adjustments

Baseline mortality adjustment: In order to account for unmeasured trends in mortality unrelated to influenza virus circulation (e.g. changes in socioeconomic status and healthcare) we standardized seasonal excess mortality rates by baseline mortality rates during summer periods, July-September, separately for each age group and country. For every season, we divided excess P&I mortality rates in that season by the 3-year average of summer mortality rates in that season, the season preceding it, and the season succeeding it. We then multiplied this ratio by the 3-year average of summer mortality rates from 1999-2001. Mortality rates in Japan were further standardized to the 3-year average of summer mortality rates in the US from 1999-2001.

Age adjustment: To generate age-adjusted excess P&I mortality estimates in seniors, we applied summer-adjusted P&I excess mortality rates for each age group (65-69, 70-74, 75-79, 80-84, 85-89) to the US population structure of 2000. This procedure was done for both Japan and the US.

Comparing excess mortality trends across periods and countries

We obtained mean excess P&I mortality rates (crude and adjusted) for the 1978-1994 and the 1995-2006 periods for 65-89 year olds in Japan and the US. We calculated the percent change in mortality rate between the two periods by subtracting the mean mortality rate from 1995-2006 by that in 1978-1994, and dividing by the mean mortality rate in 1978-1994. To test the null hypothesis that the distribution of mortality rates in 1978-1994 was identical to that in 1995-2006, we utilized Wilcoxon’s nonparametric rank-sum test (Tables S4-S5). We compared the same time periods in the US and Japan to control for potential differences in the severity of circulating influenza strains, beyond subtype.

Estimating the indirect benefits of vaccination

To compare the risk of death for persons aged 65-89 years during the vaccination program period (1978-1994) with the period when the program had been interrupted (1995-2006), we modeled seasonal excess death rates using multivariate negative binomial regression with a dummy variable for time period, controlling for age and A/H3N2 subtype dominance. We utilized a backwards stepwise selection procedure to identify possible pair-wise interaction terms. The approach was applied to both crude and adjusted excess P&I mortality rates. Vaccine mortality reduction was calculated as (1-1/RR) x 100, where RR is the exponent of the estimated dummy variable parameter for time period (during and after schoolchildren vaccination).

We also compared influenza vaccination strategies in Japan with those in the US by modeling adjusted seasonal excess P&I mortality rates (standardized to the US population structure and summer mortality rate of 2000) in both countries (Table S6). As in other models, we controlled for age and A/H3N2 subtype dominance.

Sensitivity Analyses:

As a measure of robustness, we sought to ensure that our findings were not simply the byproduct of the time periods we chose for comparison. We therefore compared excess mortality rates among Japanese and American seniors during the last ten influenza seasons of the schoolchildren vaccination program (1983-1994) with those in the first ten seasons after the program’s discontinuation (1995-2006; Table S7).

Influenza Transmission Model:

We used an age-structured transmission model to quantify the indirect protection against death provided to senior populations by vaccinating schoolchildren. A schematic of the compartmental epidemic model is given in Figure S1. Our transmission model was previously developed to study the impact of vaccination strategies against pandemic influenza [5]. We adapted the model to incorporate 15 age groups (0-4 y, 5-9 y, 10-14 y,15-19 y, 20-24 y, 25-29 y, 30-34 y, 35-39 y, 40-44y, 45-49 y, 50-54 y, 55-59 y, 60-64 y, 65-69 y, >=70 y) which were populated using Japan’s population estimates for 1978 when the schoolchildren vaccination policy was put in place. Each age group (indexed by *i*) is classified into 6 epidemiological states given by: susceptible (S_i_), latent (E_i_), symptomatic and infectious in the community (I_i_), hospitalized (H_i_), protected (P_i_), and dead (D_i_). Susceptible individuals in age group *i* are exposed to the influenza virus at the force of infection $\lambda_{i}=\sum_{j=1}^{15} \beta_{ij}\frac{I_{j}+H_{j}}{N(t)}$ where $\beta_{ij}$is the transmission rate between age groups *i* and *j* and the total population size is given by

$N(t)=\sum_{k=1}^{15} S_{k}(t)+E_{k}(t)+I_{k}(t)+H_{k}(t)+P_{k}(t)$.

The transmission rates $\beta_{ij}$ are given by qc_ij_ where q is the transmission probability per contact, which is assumed to be constant across age groups and c_ij_ are the age-specific contact rates which are modeled based on a study describing self-reported age-specific contact rates in European countries [6]. Overall, the contact rate matrix is highly assortative with higher mixing rates within each age group than between age groups [6]. Latent individuals E_i_ progress to the infectious class I_i_ at the rate k (1/k is the mean latent period of ~2 days). Infectious individuals are hospitalized at the age-specific mean rates α_i_ and recover at the mean rate γ_1_. Hospitalized individuals either recover at the constant rate γ_2_ or die from influenza at the age-specific rate δ_i_. While the age-specific hospitalization rates are adjusted using estimates of the probability of hospitalization given clinical illness by age group, the recovery rate γ_2_ is assumed to be constant across age groups for simplicity (2-4 days). Recovered individuals are assumed to remain protected for the duration of the epidemic. Infected individuals die with an age-specific mortality rates as described below. We used a simple immunity structure based on [7] with 30.6% of children (<=19 y) and 25.6% of adults (>19 y) assumed initially protected.

We simulated vaccination coverage rates among schoolchildren (5-14 y) in the range 50-90% and vaccine efficacy ranges between 30 and 70%[8, 9]. Effectively vaccinated individuals prior to the influenza season were moved to the protected class.

The system of differential equations that describes our influenza transmission model is given by

$$\begin{matrix} \frac{d S_{i}}{d t}=-\nu(t) S_{i}-\lambda_{i} S_{i} \\ \frac{d E_{i}}{d t}=\lambda_{i}(S_{i}+ V_{i}+U_{i})-k E_{i} (1) \\ \frac{d I_{i}}{d t}=k E_{i}-(\alpha_{i}+\gamma_{1}) I_{i} \\ \frac{d H_{i}}{d t}=\alpha_{i}I_{i}-( \gamma_{2}+\delta_{i}) H_{i} \\ \frac{d P_{i}}{d t}=\gamma_{1}I_{i}+ \gamma_{2}H_{i} \\ \frac{d D_{i}}{d t}=\delta_{i} H_{i} \end{matrix}$$

for $i=1,\ldots,15$.

The hospitalization and mortality rates are given by α_i_ = ($p_{H_{i}}$/(1 − $p_{H_{i}}$))γ_1_ and δ_i_ = (*CFP*_i_/(1−*CFP*_i_))γ_2_, respectively, where $p_{H_{i}}$ and *CFP*_i_ denote the probability of hospitalization given clinical illness and the probability of death following hospitalization for age group *i* were based on estimates for seasonal influenza [5]. The system of ordinary differential equations was solved numerically using Matlab (The Mathworks, Inc.) with initially 10 infectious young individuals (10-14 y).

The effective reproduction number R_e_ measures the average number of secondary cases generated by a primary infectious in a partially naïve population [10, 11]. We set the effective reproduction number in the range 1.1-1.9 by scaling the probability of transmission given contact (q) [5].

**Supplementary References:**

1. Anderson R, Minino A, Hoyert D, Rosenberg H (2001) Comparability of Cause of Death Between ICD-9 and ICD-10: Preliminary Estimates. National Vital Statistics Reports 49.

2. Sugaya N, Takeuchi Y (2005) Mass vaccination of schoolchildren against influenza and its impact on the influenza-associated mortality rate among children in Japan. Clin. Infect. Dis 41: 939-947.

3. Simonsen L, Reichert TA, Viboud C, Blackwelder WC, Taylor RJ, et al. (2005) Impact of influenza vaccination on seasonal mortality in the US elderly population. Arch. Intern. Med 165: 265-272.

4. Viboud C, Grais RF, Lafont BAP, Miller MA, Simonsen L (2005) Multinational impact of the 1968 Hong Kong influenza pandemic: evidence for a smoldering pandemic. J. Infect. Dis 192: 233-248.

5. Chowell G, Viboud C, Wang X, Bertozzi SM, Miller MA (2009) Adaptive vaccination strategies to mitigate pandemic influenza: Mexico as a case study. PLoS ONE 4: e8164.

6. Mossong J, Hens N, Jit M, Beutels P, Auranen K, et al. (2008) Social contacts and mixing patterns relevant to the spread of infectious diseases. PLoS Med 5: e74.

7. Cauchemez S, Valleron A, Boëlle P, Flahault A, Ferguson NM (2008) Estimating the impact of school closure on influenza transmission from Sentinel data. Nature 452: 750-754.

8. Hirota Y, Kaji M (2008) History of influenza vaccination programs in Japan. Vaccine 26: 6451-6454.

9. Jefferson T, Rivetti A, Harnden A, Di Pietrantonj C, Demicheli V (2008) Vaccines for preventing influenza in healthy children. Cochrane Database Syst Rev : CD004879.

10. Anderson RM, May RM, Anderson B (1992) Infectious Diseases of Humans: Dynamics and Control. Oxford University Press, USA.

11. Diekmann O, Heesterbeek JAP (2000) Mathematical Epidemiology of Infectious Diseases: Model Building, Analysis and Interpretation. 1st ed. John Wiley & Sons.
